# Acute Intermittent Porphyria in Argentina: An Update

**DOI:** 10.1155/2015/946387

**Published:** 2015-05-17

**Authors:** Gabriela Nora Cerbino, Esther Noemí Gerez, Laura Sabina Varela, Viviana Alicia Melito, Victoria Estela Parera, Alcira Batlle, María Victoria Rossetti

**Affiliations:** ^1^Centro de Investigaciones sobre Porfirinas y Porfirias (CIPYP), CONICET, Hospital de Clínicas, Universidad de Buenos Aires (UBA), 1120 Buenos Aires, Argentina; ^2^Departamento de Química Biológica, Facultad de Ciencias Exactas y Naturales, Universidad de Buenos Aires (UBA), Ciudad Universitaria, Núnez, Universidad de Buenos Aires (UBA), 1120 Buenos Aires, Argentina

## Abstract

Porphyrias are a group of metabolic diseases that arise from deficiencies in the heme biosynthetic pathway. A partial deficiency in hydroxymethylbilane synthase (HMBS) produces a hepatic disorder named Acute Intermittent Porphyria (AIP); the acute porphyria is more frequent in Argentina. In this paper we review the results obtained for 101 Argentinean AIP families and 6 AIP families from foreign neighbour countries studied at molecular level at Centro de Investigaciones sobre Porfirinas y Porfirias (CIPYP). Thirty-five different mutations were found, of which 14 were described for the first time in our population. The most prevalent type of mutations was the missense mutations (43%) followed by splice defects (26%) and small deletions (20%). An odd case of a double heterozygous presentation of AIP in a foreign family from Paraguay is discussed. Moreover, it can be noted that 38 new families were found carrying the most frequent mutation in Argentina (p.G111R), increasing to 55.66% the prevalence of this genetic change in our population and adding further support to our previous hypothesis of a founder effect for this mutation in Argentina. Identification of patients with an overt AIP is important because treatment depends on an accurate diagnosis, but more critical is the identification of asymptomatic relatives to avoid acute attacks which may progress to death.

## 1. Introduction

The porphyrias are a heterogeneous group of metabolic disorders that result from the decreased activity of a specific enzyme of the heme pathway and are characterized by the overproduction and excretion of heme intermediates in urine and/or stool and their accumulation in certain tissues [1–3].

Acute Intermittent Porphyria (AIP, OMIM 176000) is the most common of the acute hepatic porphyrias. It is an autosomal dominant disorder caused by a deficient activity of hydroxymethylbilane synthase (HMBS, EC 4.3.1.8), also referred to as porphobilinogen deaminase, producing a markedly increase in the urinary excretion of ALA and PBG. The symptoms may frequently appear at any time after puberty and are characterized by acute neurovisceral signs which include intermittent attacks of abdominal pain, constipation, vomiting, hypertension, tachycardia, fever, and various peripheral and central nervous system manifestations. Acute attacks may frequently result from exposure to diverse porphyrinogenic drugs, alcohol ingestion, reduced calories intake due to fasting or dieting, infections, and hormones which stimulate heme synthesis by ALA-synthase induction, thereby increasing the production of the porphyrin precursors ALA and PBG [4, 5].

HMBS is the third enzyme involved in heme pathway and catalyzes the head to tail condensation of four molecules of PBG to form the lineal tetrapyrrole HMB. It is encoded by a single gene localized at the chromosomal region 11q23.3. The cDNA and the entire 10 kb gene have been sequenced including the 5′ regulatory, 3′ regulatory, and intronic regions. The gene contains 15 exons and 2 distinct promoters that generate housekeeping and erythroid transcripts by alternative splicing and cDNAs encoding the 44-kD housekeeping and the 42-kD erythroid-specific isoenzymes, which have been isolated and characterized [6].

AIP is the most common acute porphyria in our country [7]. It is an autosomal dominant disorder with incomplete penetrance although some cases of homozygosity or double heterozygosity have been described, in most cases associated with childhood and more severe manifestations [8–21]. The identification of asymptomatic heterozygotes in families with affected individuals is essential for their counselling to avoid specific precipitating factors, but as the enzyme assay is only about 80% accurate [1], the use of molecular techniques to identify specific mutations in the* HMBS* gene is essential for accurate diagnosis of affected members in AIP families [7].

To date about 390 different mutations have been identified in the* HMBS* gene causing AIP (Human Gene Mutation Database HGMD, http://www.hgmd.cf.ac.uk/ac/index.php); most of them were either private or found in a few unrelated families, showing the molecular heterogeneity of AIP.

We review here all the mutations found in 101 Argentinean and 6 foreign AIP families and studied during the last 20 years at CIPYP. Four new mutations and 31 already described genetic changes were found; some of them were detected for the first time in our population. It must be highlighted that 59 unrelated families carry the same mutation, p.G111R (55.66%), increasing its number with respect to that previously found [22], suggesting a founder effect for this genetic change as has been described for different mutations in other populations [23–25].

## 2. Materials and Methods

### 2.1. Patients

Informed consent was obtained from all patients following the standards of UNESCO Declarations-DD.HH Genome and Genetic Data (http://www.unesco.org/shs/ethics), Declaration of Helsinki was taken into consideration, and the study was approved by the Institutional Research Ethics Committee of the CIPYP, National Scientific and Technical Research Council (CONICET), University of Buenos Aires (UBA).

From March 1994 to July 2014, 106 unrelated Argentinean families were studied at biochemical and molecular level. All patients had current symptoms of AIP and the diagnosis was made on the basis of their clinical history of at least one acute attack associated with increased excretion of ALA and PBG in urine and reduced HMBS activity in red blood cells [1]. The final diagnosis of the patients was established by genetic studies. Unrelatedness was determined by family inquiries.

### 2.2. Identification of Mutations

#### 2.2.1. DNA Isolation and HMBS Amplification

Genomic DNA was extracted from peripheral blood collected in EDTA using the commercial kit illustra^TM^ blood genomicPrep Mini Spin Kit (GE Healthcare). Mutational analysis was performed amplifying the promoters, all exons, and the intron/exon boundaries of the* HMBS* gene by PCR using the specific primers shown in Table 1. Promoter regions and genomic sequence from exon 3 to noncoding exon 15 were amplified in only two fragments using Platinum Taq DNA Polymerase High Fidelity enzyme (Invitrogen by Life Technologies). Alternatively, exons 3 to 15 and their flanking intron regions were amplified in 5 fragments as indicated (see Supplementary Material available online at http://dx.doi.org/10.1155/2015/946387), employing recombinant Taq DNA Polymerase (Invitrogen by Life Technologies).

#### 2.2.2. RT PCR

RNA was isolated from the leukocytes using the commercial kit Ribo Pure–Blood (Ambion) and reverse transcribed with M-MLV Reverse Transcriptase and Oligo (dT)_12–18_ primers (Invitrogen), according to manufacturer's instructions. The HMBS cDNA was amplified with the primers Fc (5′ aaagcctgtttaccaaggagc 3′)–Rc (5′ caccaccagctccaagatgt 3′).

All PCR products were checked in 1.5% agarose gel.

#### 2.2.3. Sequencing Analysis

The amplified products were purified with the Bioneer Accuprep PCR Purification Kit (Bioneer) or QIAquick PCR/Gel Purification Kit (QIAGEN) and were automatically sequenced by Macrogen (Macrogen Inc., Gangseo-gu, Seoul, Korea, ABI3730XL, Macrogen). The sequencing primers are listed in Supplementary Material. All mutations were confirmed by sequencing both DNA strands of at least two different PCR products. To validate the new mutations, their absence in 50 control individuals has been performed. Nucleotides were numbered according to the cDNA sequence for the housekeeping isoform of HMBS transcript variant 1 (GenBank Accession NM_000190.3) in which the A of the ATG initiation codon was numbered as 1.

#### 2.2.4. Databases

The Human Gene Mutation Database (http://www.hgmd.cf.ac.uk/) was used for information about reported mutations in the* HMBS* gene.

## 3. Results

At present 177 AIP families (299 affected individuals) were diagnosed at CIPYP. Of them, 107 were also studied at molecular level and results for 48 families were already described [7, 28, 29, 34]. In the last 10 years 58 new families were also biochemically diagnosed as AIP and molecular analysis revealed 19 mutations, 4 new and 15 already reported of which 7 were described for the first time in our population (Table 1).

**Figure 1:**
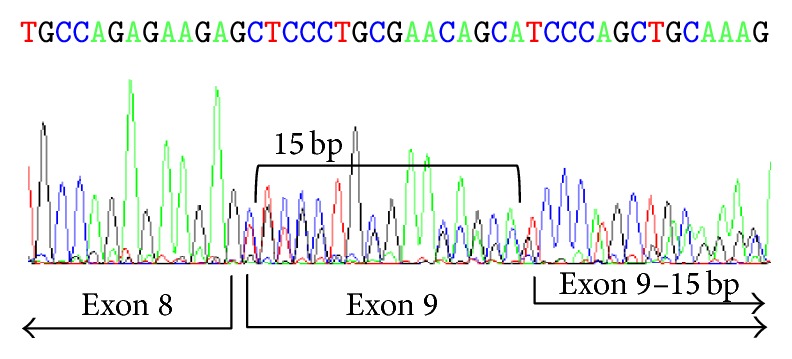
Electropherogram showing c.423-2A>G mutation RT-PCR product.

From the novel mutations, two were splice site mutations at acceptor splice sites. One was an A to G transition in the penultimate base of intron 8 leading to the in-frame deletion of 15 bp with the loss of the first 5 amino acids of exon 9 (c.423-2A>G) by the use of a cryptic site (Figure 1). The other was also an A to G transition in the last base of intron 14 (c.913-1G>A) predicting the skipping of exon 15.

**Figure 2:**
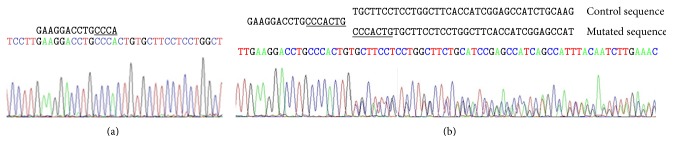
Electropherograms showing (a) control sequence; (b) c.301_307dupCCCACTG mutation; the duplicated sequence is underlined.

In another family, an out of frame new duplication of 7 bp in exon 7 which generates a stop codon 17 bp upstream (c. 301_307dupCCCACTG) was found (Figure 2).

**Figure 3:**
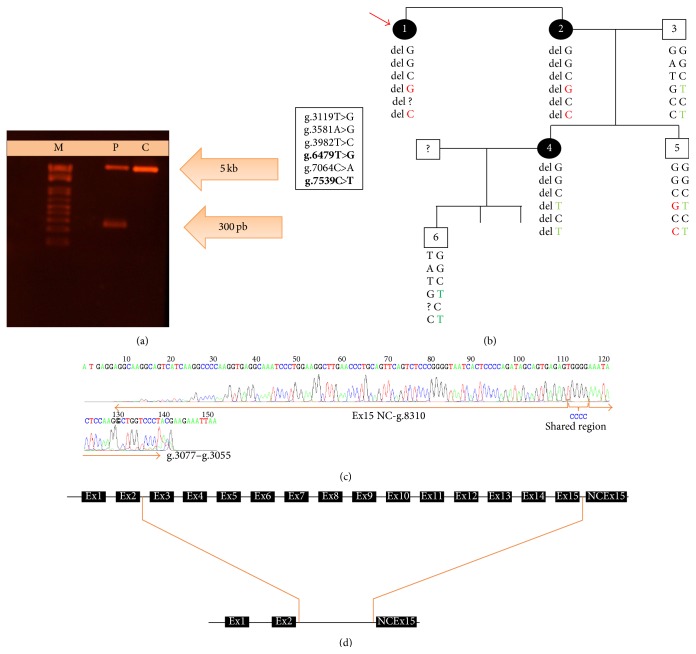
Gross deletion of 5228 bp. (a) PCR product of patient (P), control individual (C), M: 1 kb marker; (b) family SNPs analysis; (c) electropherogram of the 300 bp band showing the shared region between intron 2 and noncodifying exon 15; (d) scheme showing the deleted region.

In another two families, 2 bands were found when* HMBS* gene was amplified, one of the expected size and another of 300 bp (Figure 3(a)). The sequencing of the small purified band revealed a large deletion of 5228 bp spanning from intron 2 to noncoding exon 15 (Figures 3(c) and 3(d)).

The other 15 mutations were previously described pointing that 7 of them were identified for the first time in our population (Table 1).

Of note are the results obtained for a foreign family from Paraguay. Two female symptomatic members carried two already described mutations. One was a splice site mutation in the last base of intron 12 inherited from the mother (c.772-1G>A) which leads to exon 13 skipping [39]. The other was a point mutation (c.962G>A) in exon 15 which produces an amino acid change (p.R321H) and was inherited from the father [22]. Another asymptomatic sister carried only this last mutation. The biochemical values and molecular results for this family are shown in Table 2.

In addition, 38 new families carry the p.G111R mutation previously described for another 21 unrelated families [7] ascending the number of unrelated Argentinean families that carry this mutation to 59 (55.66%) adding further support to our previous hypothesis of a founder effect [28].

## 4. Discussion

During the last 20 years, 35 different mutations were found: 14 described in Argentina for the first time and 21 already reported for other populations. These 35 genetic changes include 15 missense mutations, 9 splice defects, 7 small deletions, 2 small insertions, one gross deletion, and 1duplication.

One of the splice site mutations is located in the −2 position of the acceptor splice site of intron 8 (c.423-2A>G, GENBANK HM856802) leading to the in-frame deletion of 15 bp (Figure 1). The same result has been found for a point mutation in the last base of intron 8 already described for another Argentinean family [34].

The other novel splice site mutation was an A to G transition in the last base of intron 14 (c.913-1G>A). Although no sample was available to carry out RT-PCR studies, as this base is 100% conserved in the consensus splice site, it is very likely that this substitution leads to exon 15 skipping as it has been described by Puy et al. for a different mutation affecting the same acceptor splice site [39].

The novel frameshift mutation (Figure 2), c.301_307dupCCCACTG (GENBANK HQ7315521), introduces a premature stop codon at exon 8 so the transcript codified by this allele is most likely to be degraded by the nonsense-mediated mRNA decay (NMD) [42]. This mutation has been found in two unrelated Argentinean families (Table 1).

In two families, two bands were found when PCR product was run in an agarose gel, one of the expected size and another of 300 bp (Figure 3(a)). When these were sequenced the large one did not show any genetic change but it showed an apparent homozygosis of the 6 variable SNPs in the studied population (g.3119T>G, g.3581A>G, g.3982T>C, g.6479T>G, g.7064C>A, g.7539C>T) Cerbino [43]. However, as it is shown in Figure 3(b), for two of these SNPs, (g.6479T>G and g.7539C>T), the proband and her sister carry the same allelic variant (6479 G and 7539 C), but the symptomatic daughter of one of them carries another allelic variant (6479 T and 7539 T) inherited from her father. The analysis of the smaller band indicated that this corresponded to the other allele with a large deletion of 5228 bp spanning from intron 2 to noncoding exon 15. As indicated in Figures 3(c) and 3(d), positions g.3078_g.3081 and g.8306_g.8309 shared the same region (CCCC) so it was impossible to determine the breakpoint of the deletion. Only another gross deletion of 4620 bp but including promoter and exon 1 has been described by Di Pierro et al. [44].

In the family from Paraguay two double heterozygotes relatives were found. The proband of this family has been diagnosed as AIP 15 years ago carrying one reported mutation, c.772-1G>A, a splice site mutation which leads to exon 13 skipping [39]. When three of her daughters came for diagnosis, it was found that one of them, asymptomatic but with a HMBS activity reduced to 50% of control value, did not carry the family mutation. A reexamination of mother DNA confirmed only the previous mutation but a more extensive study of her daughters indicated that two of them carry two mutations, the maternal mutation and another previously described missense mutation, p.R321H [22], inherited from their father. The asymptomatic daughter carries this last genetic change. It is interesting to note that these patients developed the symptomatology recently (23 and 26 years old) and only one of them occasionally suffered from some abdominal pain. In most of homozygous or double heterozygous AIP reported cases the enzyme activity was severely reduced and the symptomatology was developed at an early age with severe neurological manifestations [13, 15, 16, 18–21]. In several of these cases essential arginine residues for enzyme activity are affected [45–47]. In these new cases the mutations found do not seem to be essential for enzyme expression or activity. This is likely true for the missense mutation which affects a nonconserved arginine residue in exon 15 located in the 29-residue insert between strands *β*3 and *α*2 in the domain 3 only present in the human enzyme sequence [47]. However, the splice site mutation leads to exon 13 skipping [39], an exon where the residue Cys^261^ is located to which the essential DPM (dipyrromethane) cofactor is bound [47]. Nevertheless this combined heterozygous genotype does not seem to have a more serious impact on HMBS activity than in the heterozygous form, since the activity of the three sisters is approximately the same. These results highlighted the importance of carrying out a complete genotype investigation of family members of a known carrier.

Finally 38 new families carrying the frequent p.G111R mutation have been characterized leading to 59 (55.66%) the number of Argentinean families carrying this genetic change. The other mutations were found in one or in a few families. Efforts were made to know if the AIP patients showing this mutation have a common ancestral origin. Detailed pedigrees were unavailable, because either relatives of many of the patients were dead or the relatives themselves had limited knowledge of their families ancestry. Since the p.G111R mutation occurs at a hot spot CpG dinucleotide, it can be possible that the mutation had been originated several times independently. However, a preliminary analysis of four intragenic and four flanking DNA polymorphic markers indicated that all tested patients with p.G111R mutation (7/21) had at least one common allele for all intragenic and flanking markers. Argentinean AIP patients with other mutations had different alleles for the markers [28]. These previous results had suggested that individuals carrying this mutation were most likely related. Extended haplotype analysis on a large group of families with the p.G111R mutation and their relatives add further evidence to our previous hypothesis about a founder effect for this mutation in the Argentinean population [43]. Microsatellite studies in these families are being carried out.

## 5. Conclusions

This study emphasizes the molecular heterogeneity of AIP and the importance of molecular techniques as the most appropriate tools for detecting and identifying specific mutations in carriers of affected families to avoid the contact with precipitating agents.

## Supplementary Material

Primers used for amplifying and sequencing the promoter, all exons and exon/intron boundaries of the HMBS gene.

## Figures and Tables

**Table 1:** All 106 families studied at biochemical and molecular level in the last 20 years at CIPYP.

Exon/intron	Mutation	Nucleotide change	Effect	Number of families (affected individuals)	First reported
E3	p.R26C	c.76C>T	26 Arg > Cys	2 (9)	[26]

I3	IVS3ds+1G>A	c.87+1G>A	5′splice site mutation, exon 3 deletion	1 (4)	[27]

E4	p.Q34P	c.101A>C	34 Gln > Pro	6 (12)	[28]
	p.Q34X	c.100C>T	34 Gln > Stop	1 (2)	[26]
	p.T35M	c.104C>T	35 Thr > Met	1 (1)	[29]
	p.Y46X	c.138C>A	46 Tyr > Stop	1 (1)	[30]

E5	p.L68fsX69	c.202_203delCT	Out of frame deletion of 2 bp	1 (1)	[31]

E6	p.L81P	c.242T>C	81 Leu > Pro	1 (3)	[14]

E7	p.G111R	c.331G>A	111 Gly > Arg	59 (171)	[32]
	p.V103fsX120	c.298_304dupCCCACTG	Out of frame duplication of 7 bp with a stop codon at +17	1 (3)	This report

I7	IVS7+1G>C	c.344+1G>C	5′splice site mutation, deletion exon 7	1 (3)	[33]

E8	p.R116W	c.346C>T	116 Arg > Trp	1 (1)	[32]

I8	IVS8as-2A>G	c.423-2A>G	3′splice site mutation, deletion 15 bp	1 (5)	This report
	IVS8as-1G>T	c.423-1G>T	3′splice site mutation, deletion of 15 bp	2 (8)	[34]

E9	p.R149Q	c.446G>A	149 Arg > Gln	1 (1)	[35]
	p.A152del	c.453_455delAGC	Del Ala 152	1 (1)	[29]

E10	p.R173Q	c.518G>A	173 Arg > Gln	1 (4)	[36]
	p.R173W	c.517C>T	173 Arg > Trp	3 (4)	[37]
	p.R201W	c.601C>T	201 Arg > Trp	1 (1)	[38]
	p.Q204X	c.610C>T	204 Gln > Stop	1 (3)	[37]

I10	IVS10ds-1G>T	c.612G>T	Deletion of 3 aa in exon 10	3 (8)	[35]

E12	p.V221fsX242	c.665insA	Out of frame insertion of A at 665	1 (3)	[28]
	p.T243fsX249	c.728_729delCT	Out of frame deletion of 2 bp at 728-729	1 (5)	[28]

I12	IVS12ds+1G>A	c.771+1G>A	5′splice site mutation, deletion of exon 12	1 (1)	[28]
	IVS12as-1G>A	c.772-1G>A	3′splice site mutation, deletion of exon 13	1 (3)	[39]

E13	p.K272fsX287	c.815_818delAGGA	Out of frame deletion of 4 bp at 815	1 (3)	[28]

E14	p.G281del	c.841_843delGGA	In-frame deletion of GGA at 841	2 (7)	[28]

I14	IVS14-2A>G	c.913-2A>G	3′splice site mutation, deletion exon 15	1 (1)	[40]
	IVS14as-1G>A	c.913-1G>A	3′splice site mutation, deletion of exon 15	1 (1)	This report

E15	p.H301fsX306	c.913insC	Out of frame insertion of C at 913	2 (5)	[41]
	p.V315fsX328	c.948delA	Out of frame deletion of A at 948	1 (2)	[28]
	p.L329fsX341	c.985delTTGGCTGCCCAG	In-frame deletion of 329 LAAQ	1 (5)	[28]
	p.R321H	c.962G>A	321 Arg > His	1 (4)	[22]
	p.G335S	c.1003G>A	335 Gly > Ser	1 (4)	[28]

	g.3078_8306del5228bp		Deletion of 5228 bp from intron 2 to intron 15	2 (7)	This report

**Table 2:** Biochemical data and mutation status of the family from Paraguay.

Patient	Age	ALA mg/24 h	PBG mg/24 h	Porph. *μ*g/24 h	PPI*λ*: 619 nm	HMBS activity	Mutation status
Proband	38	2.7	8.7	188	1.80	44.72	c.772-1 G>A
Mother	67	—	—	—	1.85	46.24	c.772-1 G>A
Husband	62	—	—	—	1.00	61.20	p.R321H
Daughter	23	6.6	32.5	589	1.23	58.06	c.772-1 G>A/p.R321H
Daughter	26	6.1	24.7	782	1.29	31.75	c.772-1 G>A/p.R321H
Daughter	30	1.0	1.2	37	1.00	44.77	p.R321H

Age in years at diagnosis. Porph.: porphyrins.

Normal values: ALA: ≤4 mg/24 h: PBG: ≤2 mg/24 h; porphyrins: 2–250 *μ*g/24 h; Plasma Porphyrin Index (PPI) ≤1.30 (*λ*: 619); HMBS activity: 84.51 ± 11.96 U/ml GR (F); 73.13 ± 13.62 U/ml GR (M).
