# Supplementary material for: Acute Intermittent Porphyria in Argentina: An Update
Source: Biomed Res Int. 2015 May 17;2015:946387. doi: 10.1155/2015/946387 (PMC4449928; doi:10.1155/2015/946387)
Supplement: Supplementary file 1 — Primers used for amplifying and sequencing the promoter, all exons and exon/intron boundaries of the HMBS gene. [file 946387.f1.pdf]

Primer	Amplified Region	Sequence of the primer (5´→ 3´)
**PR3**	Promotor, Ex 1, Intron 1 (4200 bp)	TGCTCCCACTTCAGTTACTTGTCTTTA
**PR4**		CTGCGGGTAGAGATTTGGATTAGTCCTG
**LR3**	Exon 3 to Exon 15 (5.500 bp)	AAGGGACCAGCCTTGGAGTATTTCCCCACTC
**LR4**		GTTCCTATCTTCCCGCCAACTCCACACGC
**PBGF3800**	Exon 3 to Exon 6 (1600 bp)	GTCTACTCCATGTGGCAT
**EP48**		AGACCTAGCATACTAGGG
**AD76**	Exon 7 (240 bp)	CCCTAGGCTCCACCACTGAAG
**EP77**		AGGGTCAGGCCCCAAAGGGAAAGG
**AD8**	Exon 8 to Exon 9 (400 bp)	CGAGAGAGAATAGAGGTGATC
**EP12**		TTGTCTTTTTCCTTGGCTG
**AD10**	Exon 10 to Exon13 (1600 bp)	TCAGGCAGAGGGAACCGCACGA
**AD11**		AGAAGGTTGTGAGACAAGCTTC
**AD12.1**	Exon 12 to Exon 15 (960 bp)	ATGCTTTGCGCCATTGGTTGG
**EP15.2**		GCACTGGACAGCAGCAAC

Primers used for amplifying the promoter, all exons and intron boundaries of the HMBS gene.

Region	Primer	Sequence of the primer (5´→ 3´)
**Intron 2 to Intron 3**	LR3	AAGGGACCAGCCTTGGAGTATTTCCCCACTC
	Pr4	CTGCGGGTAGAGATTTGGATTAGTCCTG
**Exon 4 to 6**	EP71	CCTAACCTGTGACAGTCT
	EP48	AGACCTAGCATACTAGGG
**Exon 7**	EP77	AGGGTCAGGCCCCAAAGGGAAAGG
	AD76	CCCTAGGCTCCACCACTGAAG
**Exon 8 to 9**	AD8	CGAGAGAGAATAGAGGTGATC
	EP12	TTGTCTTTTTCCTTGGCTGC
**Exon 10 to 11**	AD10	TCAGGCAGAGGGAACCGCACGA
	AD11	AGAAGGTTGTGAGACAAGCTTC
**Exon 12 to 15**	AD12.1	ATGCTTTGCGCCATTGGTTGG
	EP15.2	GCACTGGACAGCAGCAAC

Primers used for sequencing the promoter region, all exons and exon/intron boundaries
